# Nanopore Sequencing Discloses Compositional Quality of Commercial Probiotic Feed Supplements

**DOI:** 10.1038/s41598-023-31626-4

**Published:** 2023-03-20

**Authors:** Worarat Kruasuwan, Piroon Jenjaroenpun, Tantip Arigul, Nipa Chokesajjawatee, Pimlapas Leekitcharoenphon, Suporn Foongladda, Thidathip Wongsurawat

**Affiliations:** 1 Division of Medical Bioinformatics, Research Department, Faculty of Medicine Siriraj Hospital, Mahidol University, Bangkok, Thailand; 2 Siriraj Long-Read Lab (Si-LoL), Faculty of Medicine Siriraj Hospital, Mahidol University, Bangkok, Thailand; 3 National Center for Genetic Engineering and Biotechnology (BIOTEC), 113 Thailand Science Park, Phahonyothin Road, Khlong Nueng, Khlong Luang, Pathum Thani Thailand; 4 Research Group for Genomic Epidemiology, National Food Institute, Technical University of Denmark, 2800 Kgs. Lyngby, Denmark; 5 Department of Microbiology, Faculty of Medicine Siriraj Hospital, Mahidol University, Bangkok, Thailand; 6 Department of Biomedical Informatics, University of Arkansas for Medical Sciences, Little Rock, AR USA

**Keywords:** Biotechnology, Microbiology, Molecular biology

## Abstract

The market for the application of probiotics as a livestock health improvement supplement has increased in recent years. However, most of the available products are quality-controlled using low-resolution techniques and un-curated databases, resulting in misidentification and incorrect product labels. In this work, we deployed two workflows and compared results obtained by full-length 16S rRNA genes (16S) and metagenomic (Meta) data to investigate their reliability for the microbial composition of both liquid and solid forms of animal probiotic products using Oxford Nanopore long-read-only (without short-read). Our result revealed that 16S amplicon data permits to detect the bacterial microbiota even with the low abundance in the samples. Moreover, the 16S approach has the potential to provide species-level resolution for prokaryotes but not for assessing yeast communities. Whereas, Meta data has more power to recover of high-quality metagenome-assembled genomes that enables detailed exploration of both bacterial and yeast populations, as well as antimicrobial resistance genes, and functional genes in the population. Our findings clearly demonstrate that implementing these workflows with long-read-only monitoring could be applied to assessing the quality and safety of probiotic products for animals and evaluating the quality of probiotic products on the market. This would benefit the sustained growth of the livestock probiotic industry.

## Introduction

Probiotics are live microorganisms that confer a health benefit on the host by restoring the gut microbial balance and improving the immune system and are mostly intended to serve human health^1^. Besides human health, the use of probiotics such as *Bacillus*, *Lactobacillus*, *Streptococcus*, and *Saccharomyces*, to promote animal health and growth performance has gained increasing attention since it can alleviate the need for antibiotic use in animal husbandry^2,3^. The guidance on the characterisation of microorganisms used as feed additives or as production organisms requires specific information on probiotics including taxonomic information, antimicrobial susceptibility and production, and the toxigenicity and pathogenicity of the strain should be identified^4^. A statement of quantity, benefit, and use-by date also needs to be provided in the probiotic product label^5^. Since microbial safety and benefits are known to be strain-specific, accurate identification and labelling are critical for ensuring the safety and efficacy of the probiotic product and gaining consumer trust.

Labelling discrepancies in the probiotic product due to either the absence of listed taxon or the presence of non-listed taxon have been broadly demonstrated. A study of 55 European probiotic products showed as many as 40–47% of the probiotic products were mislabelled^6^. In addition, many probiotic products were shown to contain unlisted, possibly pathogenic bacteria that may undermine the safety and quality of the product^7,8^. Inappropriate labelling such as misspelling, obsolete, or non-existing nomenclature, failure to indicate the number of live cells, and in some instances failure to yield any living culture was commonly reported^8–10^. Hence, the European Society for Pediatric Gastroenterology, Hepatology and Nutrition (ESPGHAN) Working Group for Probiotics and Prebiotics conducted a systematic review on quality assessment of the commercial probiotic products available worldwide. The working group identified the misidentification at the genus/species/strain level and contamination as worrisome issues among other quality problems i.e., incongruent number of viable cells and decreased functional properties. Majority of the probiotic products tested, regardless of the country of origin, yielded unsatisfactory results with more than one labelling inconsistency. A call for improved quality control of probiotic products including precise identification of microorganisms to the strain level has been issued^11^.

The majority of these studies have used conventional methods i.e. selective culture techniques, physiology, biochemistry, PCR identification, or used an out of date database for bacterial identification, which is time-consuming, limited to specific bacteria in a multispecies mixed product, and leads to misidentification. Recently, high-throughput next-generation sequencing is an emerging trend that attempts to accurately assess the microbial composition and any possibly pathogenic bacteria detected in the product. For example, whole-genome sequencing, microbiome, and metagenomic analysis using short-read sequencing was used to verify the microbial composition, and possible contamination of commercial probiotic products sold in the Canada, China, and United States marketplaces^12–14^. Given such approaches do not enable immediate species-level identification in a mixed microbial community, a gold standard PCR technique using strain-specific primers is required to verify the presence of certain organisms. Recently, the short-read metagenomic-based technique has been noticed to analyse probiotic supplements through both partial 16S rRNA-targeted sequencing (V3-V4)^15^ and shotgun metagenomic sequencing^14,16^. Though short-read sequences are typically classified at the family- or genus-level, greater taxonomic resolution to strain-level is of more interest in the quality assessment of probiotic products^17^. The MinION™ sequencer from Oxford Nanopore Technologies (ONT) is one of the single-molecule based sequencers that can be conducted in real-time with little equipment and is portable, enhancing its potential as a field tool for remote sites^18,19^. Since very long reads with no limitation of read length can be generated, ONT nanopore sequencer can help to resolve complex structure variants and repetitive regions in the genomes and can provide location of antimicrobial resistance (AMR) genes on mobile element or plasmid^20^ and nowadays, an alternative base-calling approach was improved by reaching the base accuracy up to > 99% (https://nanoporetech.com/accuracy). Therefore, long-read data could increase accuracy of microbial classification for both entire 16S rRNA gene and metagenome samples with bacterial mixtures which previously demonstrated in a gut microbiota study^19,21^.

Metagenomics facilitates to retrieve metagenome-assembled genomes (MAGs) with high contiguity and completeness from metagenomic data by assembling sequencing reads into contigs and grouped into single-taxon bins, can then be used for further high-accuracy taxonomic identification and gene annotation^22^. MAGs data also enables the prediction of AMR genes, virulence elements and biosynthetic gene clusters which can determine the presence of any microbial contaminant and further AMR-associated microorganism monitoring in the environment^23–25^. Long-read based MAGs have been noted to elucidate the microbial profiling in complex ecosystems, such as canine feces^26^, chicken gut^27^ and activated sludge^28^. Nevertheless, there is limited information using long-read data in probiotic products especially animal feed supplements. Given this, here, we compared two quality-checking techniques to investigate the list of microorganisms declared on the labels and any possible harm detected in animal probiotic feed-additive products available in Thailand. Two workflows, full-length 16S RNA gene and long-read metagenomics, were presented and compared. Using metagenomic data generated by ONT, we also demonstrated useful computational tools for MAGs taxonomy classification. AMR, virulence factors (VF), biosynthetic gene clusters (BGCs), and bacteriocins in probiotics products for animal were also characterised from MAGs.

## Results

### Animal probiotic product investigation workflows

**Figure 1:**
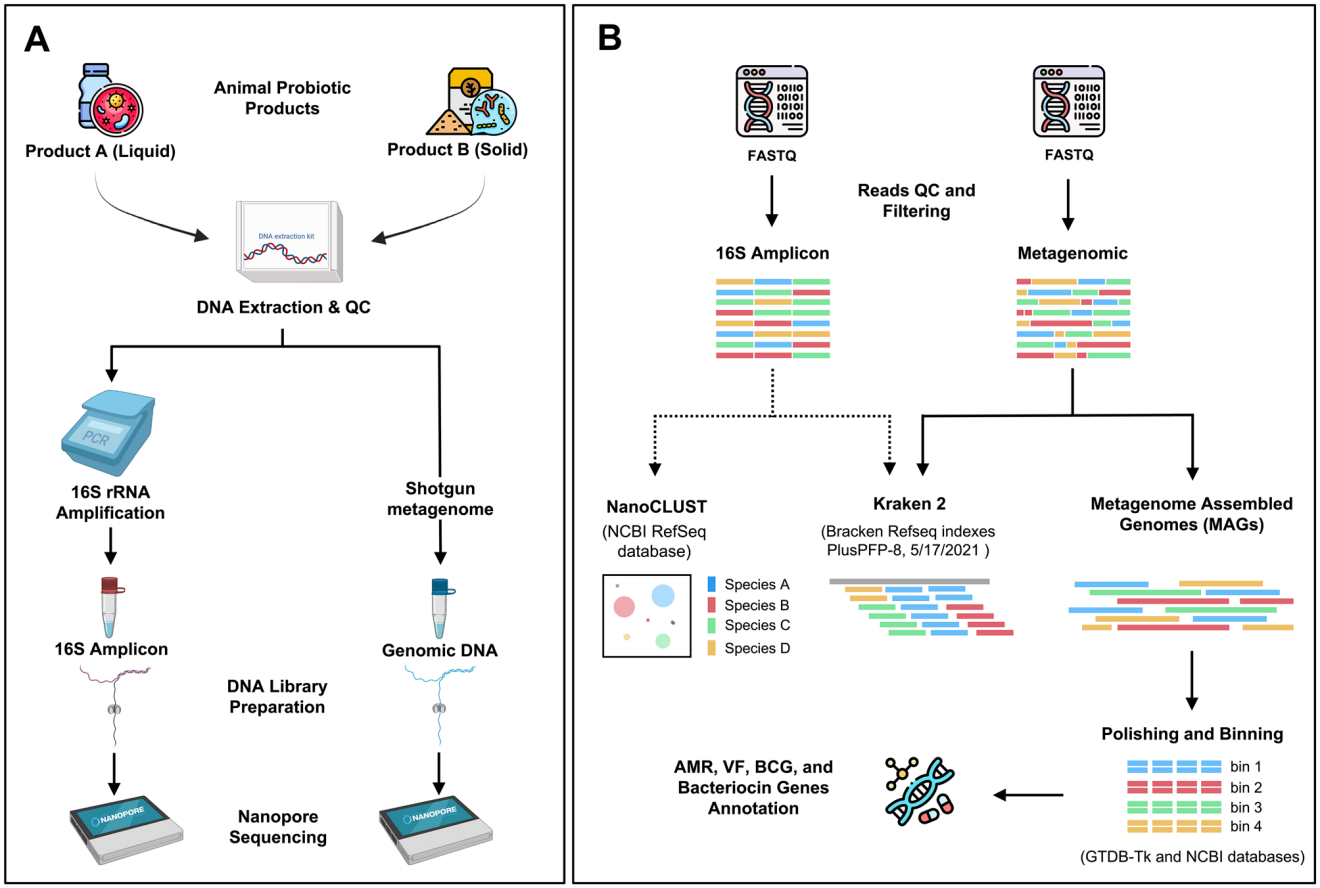
Schematic represents full-length 16S rRNA gene and metagenomic sequencing (**A**) and bioinformatic pipelines (**B**) for labelled strains investigation of animal probiotic products. Firstly, metagenomic DNA and 16S amplicon of animal probiotic products A and B were prepared and sequenced using Nanopore sequencer. Then, the nanopore reads were qualified and filtered by adapter trimming using porechop. After that, the 16S amplicon data were taxonomically identified by NanoCLUST and Kraken2 database while the metagenomic data were the taxonomically assigned using Kraken2 and assemble to the genome using the metagenome assembled-genomes (MAGs) approach. Genome taxonomy was determined using the GTDB-Tk database and only unidentified MAGs were identified by BLAST against the NCBI database. Finally, antimicrobial resistance (AMR) and function genes were predicted from metagenomic and MAGs data.

To investigate animal probiotic products available in the Thai market, both liquid and solid forms of commercial feed-additive products; designated as Product A and B, were collected and analysed. We applied long-read nanopore sequencing technology to assess detailed microbial compositional of the products as described in Fig. 1. Both full-length 16S rRNA amplicons and long-read metagenomic DNA libraries were prepared and subjected to nanopore sequencing. To display the relative abundance of each taxon in the sample, full-length 16S amplicon (16S) and metagenomic (Meta) data were taxonomically separated at the species-level using both NanoCLUST (NC) and Kraken2 (KK) tools. Probiotic genomes were reconstructed using metagenome-assembled genomes (MAGs) approach, taxonomically classification was achieved using the GTDB-Tk and NCBI database. Antimicrobial resistance (AMR) genes, virulence factor (VF), secondary metabolites biosynthetic gene cluster (BGC) and bacteriocin genes were predicted from either metagenome or MAGs data.

### Taxonomic determination using nanopore reads

**Figure 2:**
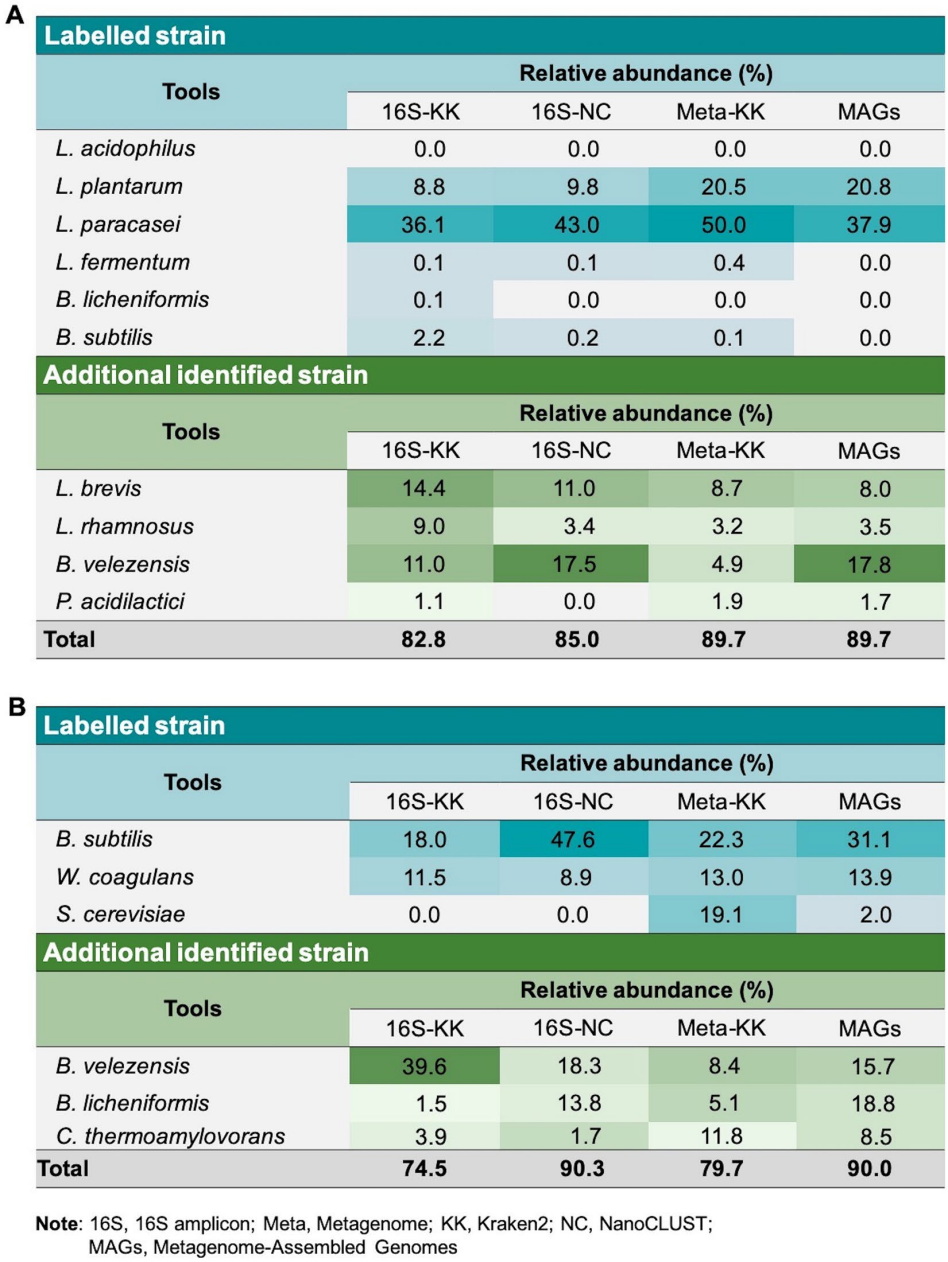
Percentage of relative abundance of labelled and additional identified strains of animal probiotic Product A (**A**) and Product B (**B**). Strains are retrieved from either 16S amplicon data against Kraken2 (16S-KK) and NanoCLUST (16S-NC) or metagenomic data against Kraken2 (Meta-KK) and metagenomic-assembled genomes (MAGs). The additional identified strains exhibit representative strains from at least three techniques. The total relative abundance values are indicated as a total of classified bacteria in both labelled and additional identified strains by each classification approach. Missing data indicate strains that could not be identified from metagenomic sequenced data.

In this study, we initially performed long-read sequencing on animal probiotic Products A and B (Table 1) to investigate microbial profiling through full-length 16S amplicon, metagenomic, and MAGs profiling. Approximately 210 Mbp and 1.1 Gbp (Product A) and 253 Mbp and 469 Mbp (Product B) of 16S amplicon and metagenomic sequence data were respectively generated. The statistics of sequencing reads in animal probiotic products are summarised in Table S1 and Fig. 2. Overall, even at low abundance (< 1%), the composition of probiotic Products A and B were found to be in near perfect agreement with what was labelled by the producer for 16S data, especially *Bacillus licheniformis* which was only found in 16S data against Kraken2 (16S-KK, Fig. 2A). Furthermore, classification of 16S data against the NCBI database using NanoCLUST (16S-NC) gave the higher number in total percentage of relative abundance in both probiotic Products A and B than Kraken2 classification (16S-KK). On the other hand, only strains with high percentage of relative abundance, such as *Lactobacillus plantarum* (20.8%) and *Lactobacillus paracasei* (37.9%) in Product A, for example, were rescued by metagenomic data (Fig. 2A). As compared to an analysis of metagenomic data against Kraken2 (Meta-KK), the assembly of metagenomic data (MAGs) yields the highest taxonomic identity (Meta-KK) (Fig. 2).

**Table 1:** Detail information of commercial probiotic feed-additives as declared on the package labels.

Product code	Source origin	Declared probiotic content	Concentration	Translational of declared information on label of product	Package size	Form	Batch number
A	Thailand	*Lactobacillus acidophilus*	110^9^ CFU/mL	Probiotic premixes for swine production	500 mL	Solution	–
		*Lactobacillus fermentum*	110^9^ CFU/mL				
		*Lactobacillus paracasei*	110^9^ CFU/mL				
		*Lactobacillus plantarum*	110^9^ CFU/mL				
		*Bacillus licheniformis*	110^9^ CFU/mL				
		*Bacillus subtilis*	110^9^ CFU/mL				
		Food additive	2 g				
B	India	*Bacillus coagulans*	≥ 410^12^ CFU/kg	Consortium of aerobic and anaerobic probiotics which reduce mortality in chicks and nursery pigs	1 kg	Powder	SYWS419H01
		*Bacillus subtilis*					
		*Saccharomyces cerevisiae*

For Product A, all tools identified *L. pararcasei* (recently reclassified as *Lacticaseibacillus pararcasei*) as the dominant taxon, followed by *L. plantarum* (recently reclassified as *Lactiplantibacillus plantarum*) whereas *L. acidophilus,* listed by the manufacturers, was not detected in the sample. Among these, other labelled bacterial species, *Lactobacillus fermentum* (recently reclassified as *Limosilactobacillus fermentum*) and *Bacillus subtilis* was recovered from 16S-KK, 16S-NC, and Meta-KK at the low relative abundant (< 1% relative abundance). In addition, *Bacillus licheniformis* was merely recovered by 16S-KK (0.1%) (Fig. 2A). For Product B, the 16S amplicon sequencing allowed to identify the bacteria in the *Bacillus* genera such as *B. subtilis* and *B. coagulans* (recently reclassified as *Weizmannia coagulans*)*,* which is listed on the product label, but the yeast *Saccharomyces cerevisiae* was not detected. For complete identification of both bacteria and yeast, the metagenome sequencing yielded more complete information (Fig. 2B). Similar to Product A, several unlisted species were identified such as *Bacillus velezensis*, *B. licheniformis* and *B. thermoamylovorans* (recently reclassified as *Caldibacillus thermoamylovorans*) as significant components by all sequencing-identification tools. Interestingly, an unlisted bacteria *B. velezensis* was identified as comprising up to 18% in Product A, and 40% in Product B, depending on the identification tools used (Fig. 2). The reason for this high prevalence either from misidentification or contamination during production process requires further investigation.

### MAGs-based taxonomic classification

**Figure 3:**
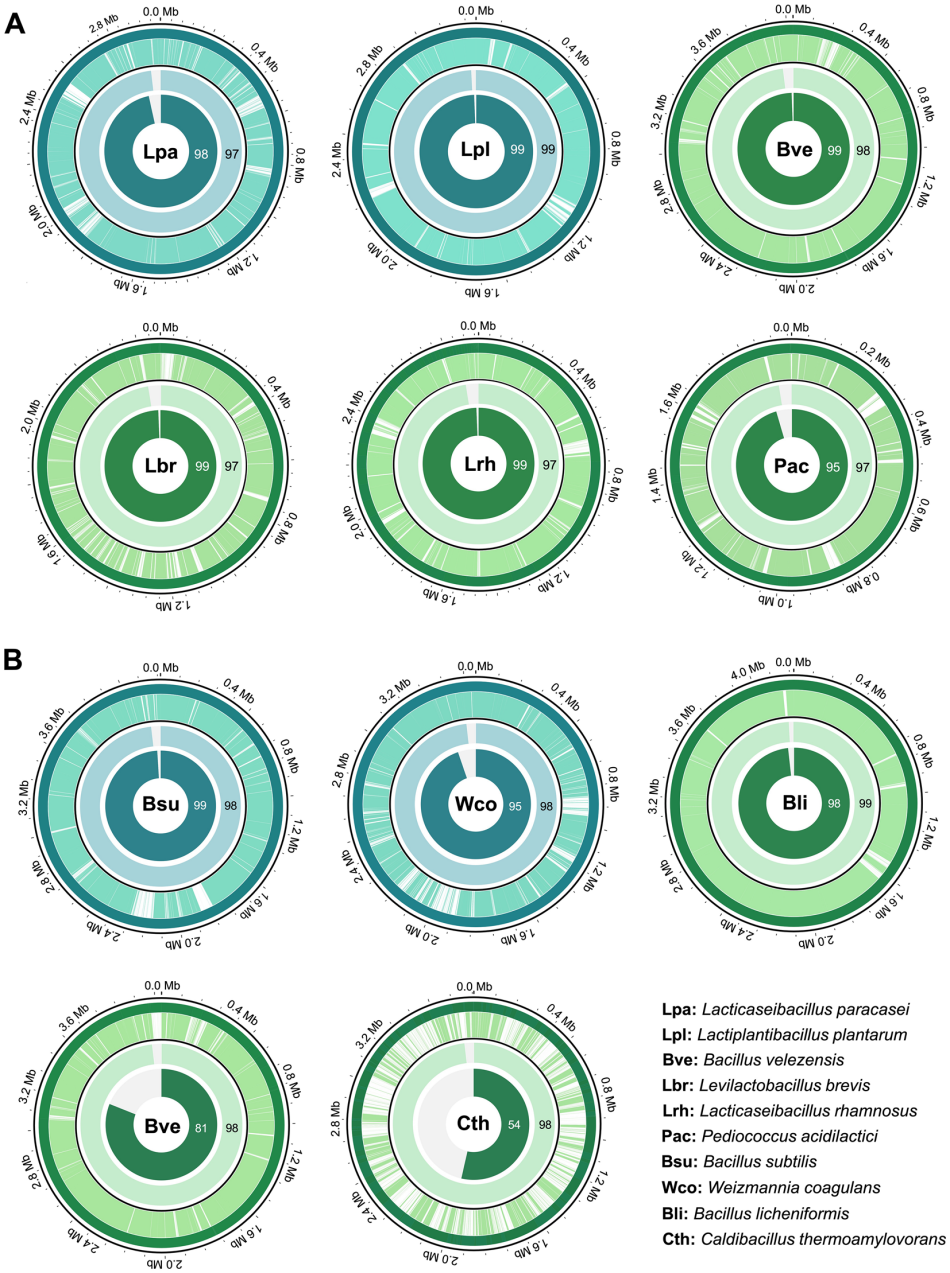
Metagenome-assembled genomes (MAGs) from animal probiotic products (**A**,**B**). Circos plot representation of features on the circular genome. Labelled and additional identified strains are shown in teal and sea green. The outer circle represents the complete genome of the closest strains. The distribution of MAG contigs all over the closest genome is located in the first inner circle. The whole-genome average nucleotide identity (ANI) percentage is calculated by FastANI and shown in the second inner circle. The percentage of completeness of the reconstructed genomes is indicated in the third inner circle.

Next, we sought to reconstruct genomes from the metagenome sequencing to classify organisms to the species-level from these samples. The MAG approach enabled the recovery of seven and four genomes of bacterial species in products A and B, respectively, with the quality of MAG by showing above 80% genome completeness and less than 10% contamination (Fig. 3, Table S4). The completeness of the genomes retrieved from MAGs ranged from 17% to 99.4% (average, 80.7%) for Product A and from 3.4% to 99% (average, 67.4%) for Product B. While genome contamination was less than 3.6% and 0.2% for all isolate genomes for Product A and B, with the exception of bin 4 of the Product B (Table S4). Furthermore, most of MAGs had a high percentage of average nucleotide identity (ANI) in the range of 97–99% in both products A and B, indicating that the MAGs obtained belonged to the same population as the isolates (Table S4).

### Abundance of AMR, VF, BGC, and bacteriocin genes in probiotic feed supplements

To evaluate the existence of antimicrobial resistance (AMR) and virulence factor (VF) genes in bacterial composition in the animal probiotic products, we used the AMR and VF screening tool, ABRicate, to annotate from either metagenomic or MAGs data against NCBI AMRFinderPlus, RestFinder, and VFDB databases. AMR analysis of metagenomic data from products A and B revealed that two and nine AMR genes (Fig. S1) respectively were distributed in animal probiotic products with ranges from 80–98.4% identity and 82–99.7% coverage (Table S5). Among these, chloramphenicol resistance gene, *cfr*(*B*), was commonly found both in products A and B. Overall, there was a higher number of AMR genes in Product B than those in Product A which were identified against antimicrobial drugs in class aminoglycosides [*ant(4')-Ib* and *aadK*], chloramphenicol (*cat4*), macrolides [*erm*(*34*)*, erm*(*D*)*,* and *mphK*] and quinolone (*qnrD1*). Whereas, the tetracycline efflux associated gene [*tet*(*L*)], was only found in Product A (Fig. S1, Table S5). Correlation of AMR analysis in metagenomics data and MAGs confirmed the present of genes involved in two (chloramphenicol and tetracycline) and three (aminoglycosides, chloramphenicol, and macrolides) classes of antimicrobial drugs with ranges from 87.3 to 99.8% identity and 98.7–100% coverage (Table 2, Fig. S1) in Product A and B, respectively. With no observation of any plasmids from labelled reconstructed genomes (Table S4), indicating that all of annotated AMR genes may locate on the chromosome. No VFs were identified in the MAGs.

**Table 2:** Occurrence of potential antimicrobial resistance (AMR) genes predicted from metagenome-assembled genomes (MAGs) of animal probiotic products.

Sequences	AMR Class	Start	Stop	Strand	Gene	%Coverage^a^	%Identity^b^	Depth^c^
Product A								
Bin1_contig_397	Chloramphenicol	1,657,165	1,658,201	–	*cfr(B)*	98.7	87.8	13.1
Bin1_contig_397	Tetracycline	3,557,900	3,559,276	+	*tet(L)*	100.0	87.3	27.2
Product B								
Bin8_contig293	Aminoglycosides	673,088	673,942	–	*aadK*	100.0	98.8	44.2
Bin9_contig227	Chloramphenicol	60,816	61,867	–	*cfr(B)*	100.0	88.3	26.3
Bin4_contig541	Macrolides	715,365	716,226	+	*erm(D)*	99.8	99.8	9.5
Bin8_contig37		1,593,377	1,594,297	–	*mph(K)*	100.0	99.0	40.8

^a^Percentage of DNA coverage (cut-off minimum DNA %coverage at 80).

^b^Percentage of DNA identity (cut-off minimum DNA %identity at 80).

Depth is an average number of mapped raw reads against putative predicted AMR gene sequences.

Biosynthetic gene cluster (BGC) encoding for secondary metabolites and bacteriocins of the MAGs were identified using antiSMASH and BAGEL4. In this work, we identified seven and ten putative BGCs in the recovered MAGs from Product A and B (Fig. S2, Table S6). Of these, three BGCs (Non-Ribosomal Peptide Synthetase or NRPS, post-translationally modified peptides or RiPPs, and trans-AT polyketide synthases or transAT-PKS) were found in both products. In the MAGs of Product A found that transAT-PKS gene clusters were the most prevalent BGCs (*n* = 3), while NRPS was dominantly found in the Product B (*n* = 8). Among these, three BGCs (arylpolyene, lanthipeptide-class-iv, and PKS) and six BGCs (batalactone, epipeptide, ladderane, lanthipeptide-class-ii, sactipeptide, and siderophore) were exclusively annotated in the either Product A or B (Fig. S2, Table S6). Furthermore, BAGEL4 revealed eight and eleven classes of bacteriocins and RiPPs from MAGs of the Product A and B. Of these, three classes (amylocyclicin, LCI, and UviB) were found in both samples. The carnocin CP52 was particularly abundant in the Product A, whereas, four (amylocyclicin, ComX2, lassopeptide and UviB) of eleven were the most abundant in the Product B (Fig. S3, Table S7).

## Discussion

There are several commercially available probiotic products available on the market, including animal probiotic products, but very few have been formally evaluated and demonstrated to contain the bacterial strains on the label by either conventional 16S rRNA gene amplification or short-read sequencing data analysis^14–16^. The emergence of long-read sequencing technology such as PacBio and Nanopore, allows the sequence of either full-length the 16S rRNA gene or long-read whole metagenome analysis which can be used to compare the sequences in public databases for microbial identification^29,30^. Although long-read sequencing has been used to monitor probiotic products for humans such as cottage cheese and paocai brine^31,32^, remarkably it has been used far less in animal feed additive products. Furthermore, recent developments in state-of-the-art software packages using metagenome data such as ATLAS, for instance, allowed to recovery of genomes from metagenome data^33^. However, most bioinformatic tools are optimised for either short-read or hybrid (short- and long-read) and a few lees implemented for Nanopore-long-read-only application^22^. Here, we modified and deployed the workflow for animal probiotics product investigation using a Nanopore long-read-only (Fig. 1). Applying Oxford Nanopore long-read sequencing and developing a bioinformatics pipeline for metagenomics study using only Nanopore long-read was firstly validated using ZymoBIOMICS™ Microbial Community DNA Standard as a control. Metagenomic profiling demonstrated the expected microbial species at anticipated abundances (Table S8 and Fig. S4), suggesting that the developed workflow is suitable for analysing long-read metagenomics data. In addition, our result found that this workflow is successful in applying both targeted 16S rRNA gene (16S) and metagenomic (Meta) data incorporated with bioinformatic tools and a suitable public database for taxonomically microbial classification. This approach also allowed metagenome-assembled genomes (MAGs) of probiotic strains to be reconstructed and used for taxonomic assignments which are consequently useful and a guideline for further application such as quality and consistency control of strains.

This work employed both 16S amplicon and metagenomic data to investigate the composition of probiotic strains in the products and demonstrates that 16S results in consistent patterns of microbial taxonomic identification when compared to metagenomics. However, using targeted 16S rRNA gene sequencing through the amplification step may introduce PCR biases in bacterial quantifying taxa resulting in underestimation of the abundance of bacterial species^34–36^. Moreover, despite the fact that targeted 16S rRNA gene sequencing is considered the gold standard for microbial species identification, only bacterial and archaea communities are detected, while fungi and viruses are excluded, with no specific ONT kits currently available for fungal community classification^37^. Conversely, even though metagenomics missed to detect the microbial species in the low abundance sample, however, MAGs enable the identification of the yeast, *Saccharomyces cerevisiae* in Product B (Fig. 2), which is overlooked using targeted 16S rRNA gene sequencing. Of note, the probiotic products used in this work (Products A and B) were listed to contain two bacterial genera (*Bacillus* and *Lactobacillus*) and only one yeast strain (*S. cerevisiae* in the Product B; Table 1). Of these, only two of the six strains listed on the label of Product A were detected using all methods, with some strains being found in low abundance within the sample. Remarkably, one strain listed as a component of the product, *L. acidophilus*, was not detected from the targeted 16S rRNA gene or metagenomic workflows, indicating that it was not present in Product A (Fig. 2). This work also demonstrated that additional strains were also present in the probiotic products. *Levilactobacillus brevis*, *Lacticaseibacillus rhamnosus*, *Bacillus velezensis* and *Pediococcus acidilactici* were all identified by at least three of the investigating methods in the Product A, while *B. velezensis*, *B. licheniformis* and *Caldibacillus thermoamylovoran* were identified in the Product B (Fig. 2). Among these, *B. velezensis* was a dominant taxon in both products which were detected in all methods used in this work. Our results confirmed the presence of this bacteria and other species by showing a high percentage of ANI value and showed a high distribution of our assembled contigs to the closest reference genome (Fig. 3, Table S4). *B. velezensis* is a member of *B. subtilis* group complex and was later reclassified as a new species based on genomic and secondary metabolites diversity^38,39^ and is considered safe and implicated as a probiotic for the animal feed-additive products for poultry^40–42^. Therefore, the lack of an updated database for safety evaluation by authorities and the highly similar prevalence of characters and sequences among this group may easily lead to misidentification and labelling discrepancies.

In this work, we used three databases, Kraken2, NCBI Refseq, and GTDB-Tk, for microbial taxonomic classification. Overall, classifying using blastn and the NCBI Refseq database in the NanoCLUST tool using 16S data (16S-NC) gave the percentage of total microbial relative abundance than Kraken2 (16S-KK) in both animal probiotic products. Generally, NanoCLUST pipeline is classified using blastn of the polished consensus through the NCBI Refseq database^43^ while Kraken2 builds its own database not only from the 16S database but also from Greengenes, SILVA, and RDP by using *k*-mer base assignments. Even though Kraken2 allowed a greater amount of reference genomic data for microbial classification, however, it was noted that Kraken2 would not identify a large proportion of reads correctly at the species level (8.93% mean absolute percentage error)^44^, particularly when genomes from various species or genera have a high level of genome similarity, as is the case in several taxonomic groups such as *Bacillus*^45^ highlighted for *B. velezensis* in this work. Compared with conventional 16S amplicon, both Kraken2 and GTDB-Tk respectively provided better consistency and a higher relative abundance of taxonomic annotation for metagenomic and MAGs of the Product A. Even two MAGs of Product A were not attributed to existing species in the GTDB-Tk database, however, the result was a consistent proportion of bacterial classification annotated from Kraken2 (Meta-KK) (Fig. 2), suggesting that both Kraken2 and GTDB-Tk are suitable to be used as reference database for bacterial identification.

Probiotics are becoming increasingly popular and recognized as generally recognized as safe (GRAS) strains for humans and animals, mostly including species of *Bacillus*, *Bifidobacterium*, *Lactobacillus*, *Streptococcus*, and the yeast *Saccharomyces boulardii*^46,47^. Even though it is classified as a GRAS strain, it is not exempt from acquiring antimicrobial resistance (AMR) genes following the FDA regulation^48^. Recently, a putative *aadk*, *cat*, *erm(D)*, *lsa(B)*, and *tet(L)* genes were characterized from five commercial *Bacillus* used as probiotic feed additives^49^. Moreover, *cfr(B)* gene, an RNA methyltransferase, was reported as a multidrug-resistant phenotype and conferred resistance to some macrolide antibiotics such as phenicols, lincosamides, oxazolidinones, pleuromutilins, and streptogramin A antibiotics found in *Bacillus* isolates from swine feces, suggesting that the possible transmission of AMR gene to host cells^50–52^. Consistent with the result of this study, *cfr(B)* gene was reconstructed in the genome of *B. velezensis* and *B. subtilis* of Products A and B, respectively. Because no plasmids were found in the MAGs of labelled probiotics in this study, the putative AMR-related genes may be intrinsic in the chromosomes of these related *Bacillus* species, indicating that such genes are less able to transmit to other bacterial species and may be safe for the host. However, further investigation on the distribution of AMR genes is needed to support this conclusion.

The rise of antibiotic-resistant bacteria due to overuse of antibiotics, both in humans and animals, has led to concern regarding the impact of AMR on global public health^53^. To decrease the use of antibiotics in animal production, the application of bacteriocins-producing probiotics for livestock has been strongly encouraged due to their immunomodulatory effect and maintenance of balance in the gastrointestinal microbiota^54^, potentially functioning as an alternative to antibiotic growth promoter^55^. For instance, various species of *Lactobacillus* including *L. acidilactici*, *L. johnsonii L. mucosae*, and *L. plantarum* isolated from the gastrointestinal tract of piglets showed significant antibacterial activities against *Escherichia coli* and *Enterobacteria*^56,57^. With the de novo assembly, it was possible to determine genes encoding bacteriocins and biosynthetic gene clusters (BGC) for secondary metabolites present within the assemblies, potentially increasing the chances of discovering novel antimicrobials using function-based metagenomic analysis as previously described^58,59^. Thus, the application of metagenomics approaches to animal probiotic products not only allows a deep understanding of microbial profiling but is also useful for identification of BGCs encoding bacteriocins which could have beneficial effects within probiotics in livestock production.

## Conclusion

In this work, we highlight a useful workflow for ensuring the safety and quality of commercial probiotic feed supplements using only Nanopore long-read. Implementation of two workflows using either 16S amplicon (16S) or metagenome (Meta) data enables animal probiotic product quality and safety assurance. The analysis of two animal probiotic products, one in liquid form and another in solid form found that both 16S and Meta data showed inconsistency in the product labels and was of special concern of the presence of antimicrobial resistance genes. Moreover, the metagenome data can be used for more in-depth analysis of the product quality/consistency/efficacy such as the production of bacteriocins and other secondary metabolites. These findings provide a guide for selecting an appropriate method for the safety assessment of animal probiotic products. In addition, using Nanopore long-read-only incorporated with our developed workflows are promising technique, and can be used as an efficient tool to monitor and ensure probiotic product quality and safety, both for the producer and regulatory body.

## Materials and methods

### Animal probiotic product samples

Two commercial animal probiotic products available in Thailand were collected on January 2022. The sample, designated as Product A and B, were used directly for metagenomic DNA extraction.

### Metagenomic DNA extraction

Metagenomic DNA was extracted from both samples of animal probiotic products. Either bacterial pellet collected from 15 mL of Product A or 10 g of Product B were extracted using the ZymoBIOMICS™ DNA miniprep Kit (D4300; Zymo Research, USA) following the modified protocol by changing from 20 to 3 min for bead-beating step to avoid DNA shearing. Next, the purity of the extracted DNA was checked by using a Nanodrop Spectrophotometer (Thermo Fisher Scientific, USA) and was quantified by a Qubit® 4.0 Fluorometer (Invitrogen, USA).

### Library preparation and nanopore sequencing

The 20 ng of metagenomic DNA was used as a template for amplifying 16S rRNA genes using the 16S Barcoding Kit (SQK-RAB204; Oxford Nanopore Technologies, UK) containing the 27F/1492R primer set. PCR amplification was performed using LongAmp™ *Taq* 2X Master Mix (New England Biolabs, UK) with the following conditions: initial denaturation at 95 °C for 1 min, 25 cycles of 95 °C for 20 s, 55 °C for 30 s, and 65 °C for 2 min, followed by a final extension at 65 °C for 5 min. The PCR product was cleaned up using AMPure XP (Beckman Coulter, USA) and a total of 100 ng DNA barcoded libraries was used for rapid adapter attachment. For the metagenome library preparation was performed using the Rapid Barcoding Sequencing Kit (SQK-RBK004; Oxford Nanopore Technologies, UK). Briefly, a total of 150 ng of metagenomic DNA was used for library preparation by cleaved with transposase enzyme to produce chemically modified ends and a barcode was added to each DNA sample, finally ligated with an adapter. The library was loaded into the R9.4.1 flow cell (FLO-MIN106; Oxford Nanopore Technologies, UK) and sequenced using MinION (Mk1C) with the default setting. ZymoBIOMICS™ Microbial Community DNA Standard (D6305; Zymo Research Corp, CA, USA) was used as a control.

### Sequence processing and taxonomic classification

Base-calling and demultiplexing were performed using Guppy v6.0.1 in the SUP (super accuracy) mode^60^. Read quality was assessed with Nanoplot v1.20.0^60^. Adapters and barcodes were removed from the reads using Porechop v0.2.4 (https://github.com/rrwick/ Porechop). The reads were filtered using NanoFilt v2.8.0^60^ with a mean quality score of > 10 with at least 1000-bp read length for 16S amplicon and a mean quality score of > 9 with at least 200-bp read length for metagenomic data. The taxonomy of 16S amplicon was assigned against both RefSeq databases (PlusPFP-8; 5/17/2021) using Kraken2 v2.1.2^44^ and NCBI databases using NanoCLUST version eb6a2c82 (committed on Dec 20, 2021)^43^. Whereas, metagenomics data were only classified to species-level against the RefSeq databases (PlusPFP-8; 5/17/2021) using Kraken2 pipeline^44^. Percentage of relative species abundance is calculated by dividing the number of species from one group by the total number of species from all groups.

### Metagenomic assembly, polishing and MAGs taxonomic identification

Metagenome-assembled genome (MAGs) (quality score of > 9 with at least 200-bp read length) was performed by assembling using metaFlye v2.9^61^. Nanopore assembly polishing was accomplished using two rounds of Racon v1.3.3 (https://github.com/lbcb-sci/Racon) and followed by one round of medaka v0.6.5 (https://github.com/nanoporetech /medaka). After that, nanopore reads were mapped to the polished nanopore assembly using minimap2 v2.24^62^. Automatic binning was performed using MetaBAT2 v2.15 with default settings^63^ and then annotated taxonomically using GTDB-Tk v1.5.1 against the GTDB R202 database (2022–04-08)^64,65^. Unmapped MAGs were further taxonomically annotated using blastn against NCBI database. The dereplicated bins were then checked for completeness and contamination using CheckM v1.0.18^66^ and MOB-suite v3.0.3 was used for plasmid typing from MAGs^67^. FastANI v1.3 was used to calculate the average nucleotide identity (ANI) of orthologous gene pairs in the scaffold and reference genomes^68^. A circular graphical display of bacterial genomic properties was done by pyCircos v0.3.0 (https://github.com/ponnhide/pyCircos).

### AMR, VF, BGC, and bacteriocin genes annotation

The genetics determinants conferring AMR and VF genes were searched by ABRicate v1.0.1 (https://github.com/tseemann/abricate)^69^ against publicly available databases; NCBI AMRFinderPlus (PRJNA313047)^70^, ResFinder (2022–05-24)^71^, and VFDB (http://www.mgc.ac.cn/VFs/)^72^. Putative biosynthetic gene clusters (BGCs) for secondary metabolites was annotated by antiSMASH v6.0^73^ with minimum contig length at 1000-bp and the minimal detection option selected so that only BGCs are detected. Annotation ribosomally synthesized and post translationally modified peptides (RiPPs) and bacteriocins on contigs were done using BAGEL4 v.1.2^74^.

## Supplementary Information

Supplementary Information 1.

Supplementary Information 2.

## Data Availability

The original contributions presented in the study are included in the article or supplementary material, further inquiries can be directed to the corresponding authors. The raw sequencing data are available at the NCBI Sequence Read Archive (SRA) under BioProject PRJNA823500^75,76^ with accession numbers SRR18682825 and SRR18682826 (16S amplicon sequencing) SRR18682824 and SRR18682823 (metagenomic sequencing).
