# Supplementary material for: Nanopore Sequencing Discloses Compositional Quality of Commercial Probiotic Feed Supplements
Source: Sci Rep. 2023 Mar 20;13:4540. doi: 10.1038/s41598-023-31626-4 (PMC10027865; doi:10.1038/s41598-023-31626-4)
Supplement: Supplementary file 1 — Supplementary Information 1. [file 41598_2023_31626_MOESM1_ESM.docx]

**Supplementary Information**

**Nanopore Sequencing Discloses Compositional Quality of**

**Commercial Probiotics Feed Supplements**

Worarat Kruasuwan^1,2^, Piroon Jenjaroenpun^1,2^, Tantip Arigul^1,2^, Nipa Chokesajjawatee^3^,
Pimlapas Leekitcharoenphon^4^, Suporn Foongladda^5^, Thidathip Wongsurawat^1,2,6*^

^1^ Division of Medical Bioinformatics, Research Department, Faculty of Medicine Siriraj Hospital, Mahidol University, Bangkok, Thailand

^2^ Siriraj Long-read Lab (Si-LoL), Faculty of Medicine Siriraj Hospital, Mahidol University, Bangkok, Thailand

^3^ National Center for Genetic Engineering and Biotechnology (BIOTEC), 113 Thailand Science Park, Phahonyothin Road, Khlong Nueng, Khlong Luang, Pathum Thani, 12120, Thailand

^4^ Technical University of Denmark, National Food Institute, Kongens Lyngby, Denmark

^5^ Department of Microbiology, Faculty of Medicine Siriraj Hospital, Mahidol University, Bangkok, Thailand

^6^ Department of Biomedical Informatics, University of Arkansas for Medical Sciences, Little Rock, AR, USA

***Correspondence:**Thidathip Wongsurawat, Ph.D.

Email: thidathip.won@mahidol.edu

**Supplementary Figures**


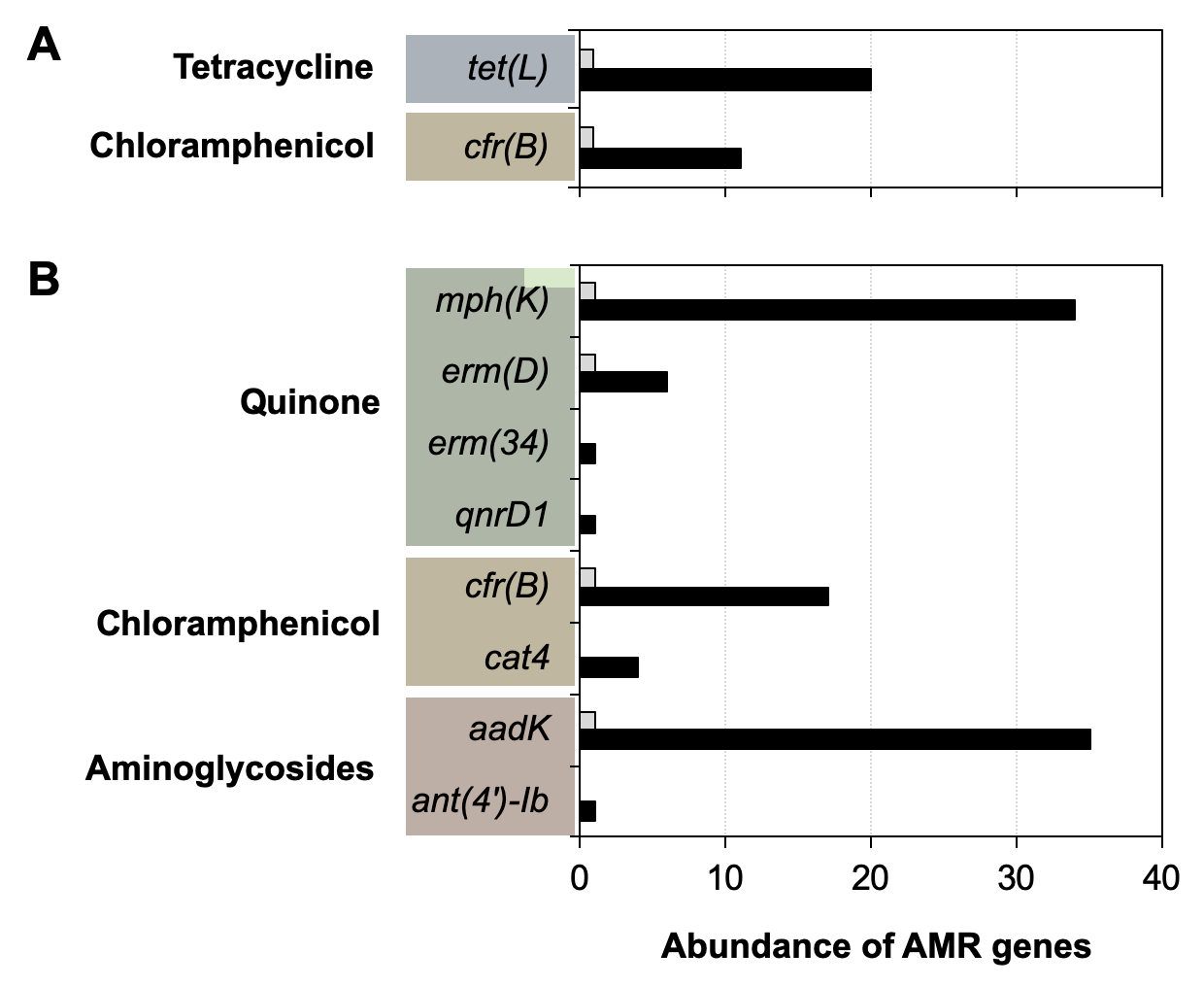


**Supplementary Figure S1.** Abundance of antimicrobial resistance (AMR) genes predicted from both metagenomic (black) and recovered MAGs (grey) of animal probiotic product A (A) and B (B) by ABRicate.


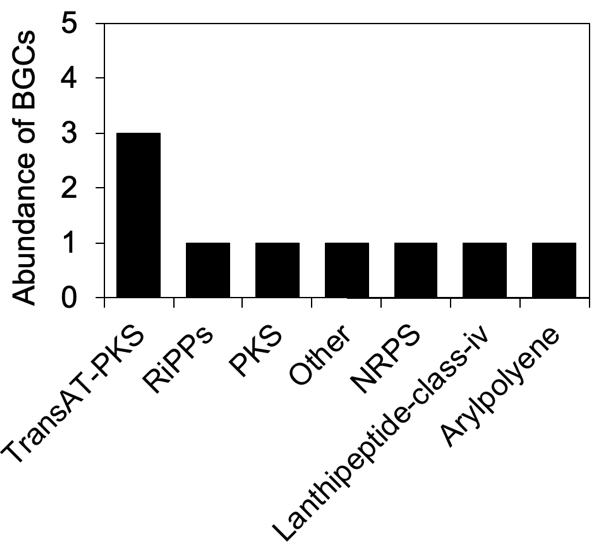
A B


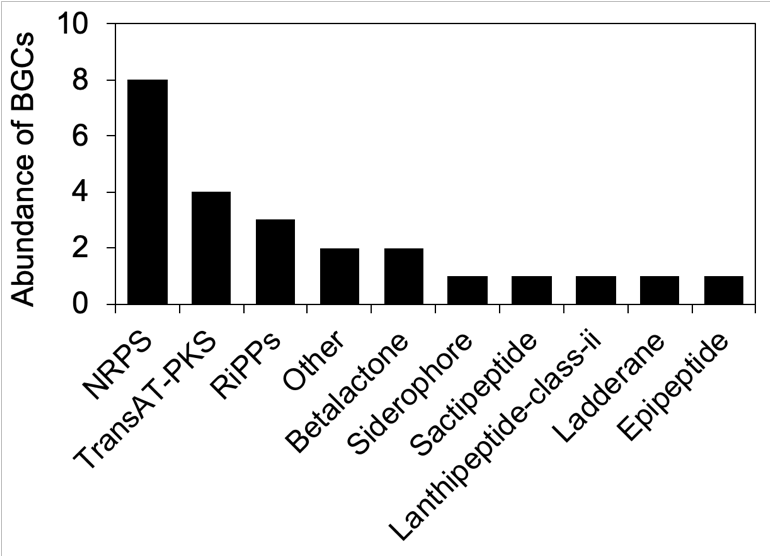


**Supplementary Figure S2.** Abundance of predicted secondary metabolite biosynthetic gene clusters (BGCs) from recovered MAGs of animal probiotic product A (A) and B (B) by antiSMASH 6.0. The description of different types of secondary metabolite clusters can be found in the glossary from antiSMASH (https://docs.antismash.secondary metabolites.org/glossary/).


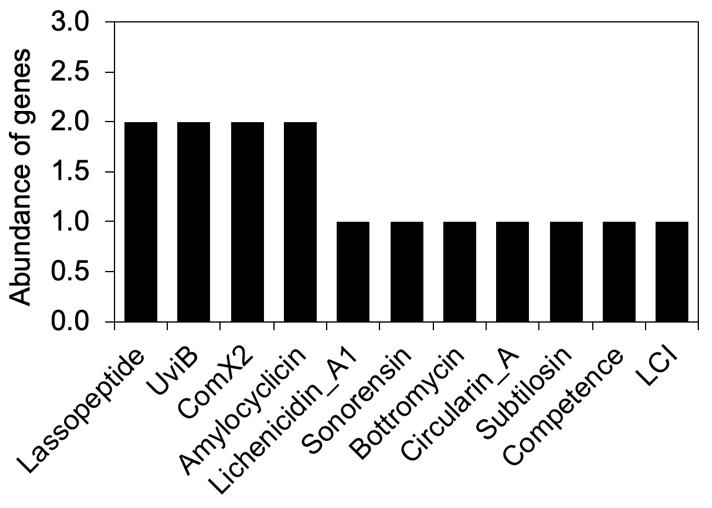

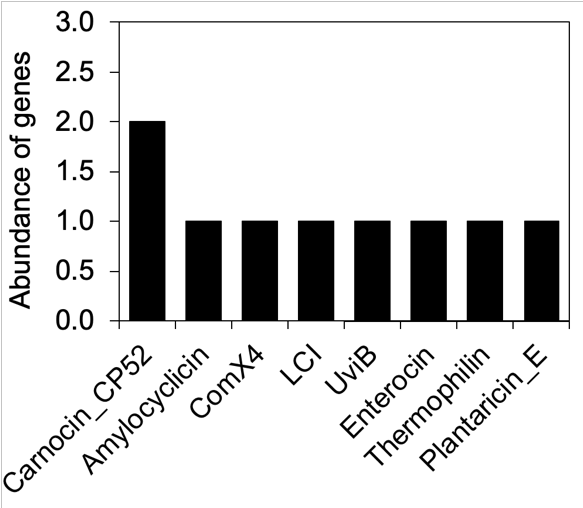
A B

**Supplementary Figure S3.** Abundance of predicted bacteriocins and other ribosomally synthesised and post-translationally modified peptides (RiPPs) genes from recovered MAGs of animal probiotic product A (A) and B (B) by BAGEL4.


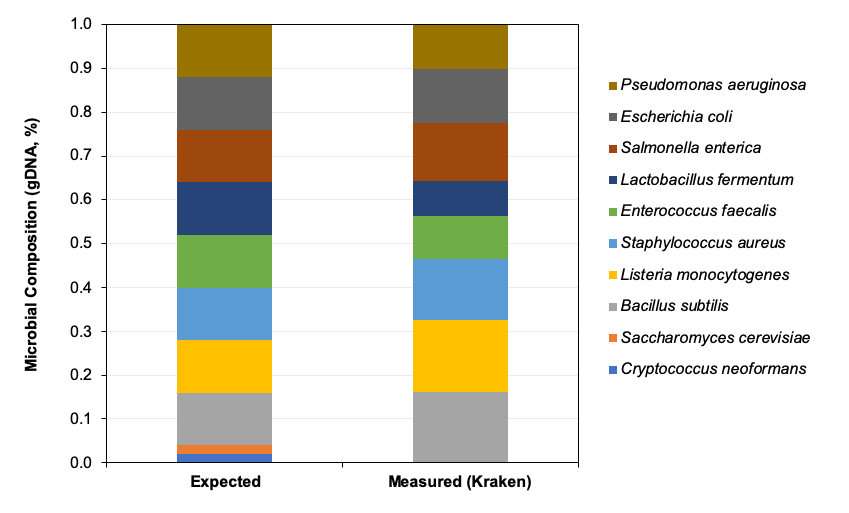


**Supplementary Figure S4.** The proportion of sequencing yield obtained from Kraken compared to the expected value for each organism in the mock community (ZymoBIOMICS™ Microbial Community DNA Standard, D6305).

**Supplementary Table Legends**

**Supplementary Table S1.** 16S amplicon, metagenomic and metagenome-assembled data statistics of animal probiotics products

**Supplementary** **Table S2.** Relative abundance (%) of taxonomically microbial classification of 16S rRNA amplicon and metagenomic data against Kraken2 (16S-KK and Meta-KK), Refseq databases in NanoCLUST tool (16S-NC) and metagenome-assembled genomes (MAGs) against GTDB-Tk databases of animal probiotic product A.

**Supplementary** **Table S3.** Relative abundance (%) of taxonomically microbial classification of 16S rRNA amplicon and metagenomic data against Kraken2 (16S-KK and Meta-KK), Refseq databases in NanoCLUST tool (16S-NC) and metagenome-assembled genomes (MAGs) against GTDB-Tk databases of animal probiotic product B.

**Supplementary** **Table S4.** Main characteristic and taxonomic classification of metagenome-assembled genomes (MAGs) from animal probiotic products.

**Supplementary Table S5.** Prediction of antimicrobial resistance (AMR) abundance in metagenomic and metagenome-assembled genomes (MAGs) from animal probiotic products.

**Supplementary Table S6.** List of secondary metabolite biosynthetic gene clusters (BGCs) detected by antiSMASH with metagenome-assembled genomes (MAGs) from animal probiotic products.

**Supplementary** **Table S7.** List of bacteriocins and RiPPs analyzed with BAGEL4 by aligning metagenome-assembled genomes (MAGs) from animal probiotic products against the core peptide databases.

**Supplementary** **Table S8.** The proportion of sequencing yield compared to the expected value for each organism in the mock community.
